# Can Long-Term Regular Practice of Physical Exercises Including Taichi Improve Finger Tapping of Patients Presenting With Mild Cognitive Impairment?

**DOI:** 10.3389/fphys.2018.01396

**Published:** 2018-09-28

**Authors:** Lingli Zhang, Yilong Zhao, Chao Shen, Le Lei, Junjie Dong, Dongchen Zou, Jun Zou, Miao Wang

**Affiliations:** ^1^School of Kinesiology, Shanghai University of Sport, Shanghai, China; ^2^Shanghai Foreign Language School, Shanghai, China; ^3^Development and Planning Office, Shanghai University of Sport, Shanghai, China

**Keywords:** mild cognitive impairment, Tai Chi, Montreal Cognitive Assessment (MoCA), dominant hand, finger tapping

## Abstract

**Background:** Mild cognitive impairment (MCI) is a brain disease with both anatomical and functional alterations. There is clear evidence that individuals that are diagnosed with MCI have a high risk to develop dementia in the next 2–5 years compared to an age-matched population with a non-MCI diagnosis. The present study aimed to investigate whether the finger tapping frequency of patients with MCI was different from that of healthy individuals without MCI, and whether Tai Chi, a traditional Chinese movement discipline, could improve the finger tapping frequency of MCI patients.

**Methods:** The study population consisted of subjects of ≥50 years of age. Group one included 40 subjects without exercise habits from communities of Yangpu District in Shanghai, and group two included 60 subjects from a Tai Chi class in Shanghai Elderly University of Huangpu District. The Montreal Cognitive Assessment (MoCA) and a finger tapping test were conducted to assess the finger tapping frequency of all subjects.

**Results:** The MoCA score of MCI subjects was significantly lower compared to subjects without MCI (*P* < 0.01), and was not influenced by age, weight, or height. The finger tapping frequency of MCI subjects’ left hands was significantly lower compared to that of healthy subjects without MCI (*P* < 0.01), and a similar trend was observed for the subjects’ right hand. The MoCA score of MCI subjects in the Tai Chi class was significantly lower than that of healthy subjects without MCI (*P* < 0.01), which was not influenced by age, weight or height. The finger tapping frequency of MCI subjects’ right hands was lower compared to that of healthy subjects in the Tai Chi class without MCI (*P* < 0.05), but no significant difference regarding the finger tapping frequency of the left hand was observed.

**Conclusion:** These findings suggested that finger tapping frequency of MCI subjects was significantly lower compared to normal subjects without MCI, and long-term Tai Chi exercise could reduce this significant difference. Moreover, there was no significant difference between groups for the subjects’ non-dominant (left) hand.

## Introduction

Mild cognitive impairment (MCI) is a brain disease with both anatomical and functional alterations, and is an intermediate clinical stage between normal cognitive aging and mild dementia. It impairs cognition of elderly individuals and reduces their reaction time, but acquired cognitive deficiency has no significant effect on the functional activities of daily living (Alzheimer, 2015), and so it is not typically diagnosed (Chen et al., 2017). Patients with MCI have an increased risk for developing Alzheimer’s disease (AD) (Beheshti et al., 2017). Patients with serious MCI over time gradually develop AD, but the progression of the disease across patients varies tremendously (Liao et al., 2016). There is clear evidence that individuals that are diagnosed with MCI have a high risk to develop dementia in the next 2–5 years compared to age-matched population with no MCI diagnosis (Shah et al., 2000; Farias et al., 2005). MCI can be seen as a transitional stage between normal aging and dementia where a subject can continue his/her daily activities (Dimitriadis et al., 2018). Detection of MCI in patients might contribute to an earlier detection of AD, which is of enormous healthcare importance, as early detection could slow the progression of the disease by the timely application of appropriate treatments (Zurron et al., 2018). MCI in study participants was determined by the Montreal Cognitive Assessment (MoCA), which is a 10 min, 30-point cognitive screening test designed to assist health professionals in the detection of MCI in patients scoring between 24 and 30 points on the Mini-Mental State Examination (MMSE).

Touch perception in the finger is an indispensable part of fine motor control, which helps contribute to the sense of self, to effective communication, and a clear perception of the world (Tan et al., 2014). Hand functions include percussion, swinging, grasping, pinching, pulling and pushing, and others. Compared with other neurologically actuated motor tasks, finger tapping has the advantage of limited inertial and intersegmental interactions that help to reduce bio-mechanical influences on body movement (Liu et al., 2006). The ability and frequency of finger tapping of an individual is a key indicator of neuromuscular integrity (Collyer et al., 1994). It has been previously shown for handedness (Aoki et al., 2016), for some individual differences in skill acquisition (Ackerman and Cianciolo, 2000), and in clinical neurological examinations (Stamatakis et al., 2013).

Repetitive rapid finger tapping is a common test of fine motor control of the upper extremities. Previous studies have proved that index finger tapping frequency is significantly correlated with the Lind-mark hand function assessment criteria score and it plays an important role in hand function evaluation in patients with stroke (Zhang L. et al., 2014). Finger tapping is usually used to assess the movement of bradykinesia patients with Parkinson’s disease (Lee et al., 2016). Moreover, a finger tapping test has been applied to measure the performance validity in most of the standard neuropsychological evaluations (Axelrod et al., 2014). The computerized finger tapping test is highly efficient and precise in evaluating finger tapping speed as well as in the measurement of potential kinetic utility in research and clinical studies of motor performance (Hubel et al., 2013). Normal finger tapping requires the functional integrity of the corticospinal tract, cerebellar motor circuitry, and proprioceptive pathways.

In the present study, we needed a quantitative indicator to identify the difference in movement control between MCI patients and healthy individuals. We thus examined the finger tapping of subjects to determine whether deterioration of finger tapping frequency in MCI patients with long-term regular practice Tai Chi training was reduced compared to non-trained control MCI subjects.

## Materials and Methods

### Ethics Statement

The Ethics Committee of the Shanghai University of Sport Human Subjects Research Review Committee granted the ethical approval for this study (Shanghai, People’s Public of China, Approval Number: 2016032). The study followed the protocols approved by the committee, and the subjects’ confidentiality was strictly maintained throughout the study. No potential conflicts of interest exist.

### Participants

The study population including 100 subjects of 50 years of age or more was divided into two groups. Group one consisted of 40 subjects (14 males and 26 females) without exercise habits chosen from Heishan community, Guohe community and Shanghai University of Sport community of Yangpu District in Shanghai from October 2016 to February 2017. Of these, 20 subjects (7 males and 13 females) were diagnosed with MCI and 20 (7 males and 13 females) were without MCI. Group two included 60 subjects (26 males and 34 females) from a Tai Chi class in Shanghai Elderly University of Huangpu District, recruited from October to December, 2016. Of the 60 subjects, 30 subjects were diagnosed with MCI and 30 were healthy controls. Subjects in group two performed two and half hours of Tai Chi exercise twice a week in a Tai Chi class, and they had been trained for more than 18 months. All participants in this study were right-handed. All potential subjects provided their consent prior to the testing period.

Exclusion criteria:

(1) Participants with acute and chronic diseases affecting the brain, for example central nerve damage, peripheral nerve damage, Parkinson’s disease and so on;
(2) Participants with dysfunctions of motor and sensory neurons, reflex action, among other functions, and in the upper body;
(3) Participants with difficulty in listening comprehension and cooperation;
(4) Left-handed participants;
(5) Participants who were athletes when they were young.

The investigators consulted the teachers of a Tai Chi class in Shanghai Elderly University and the managers of the Heishan community, Guohe community and Shanghai University of Sports community, and have chosen eligible subjects. We contacted the subjects, explained the aim and procedure, and invited them to take part in the study. All the subjects who agreed to participate signed the informed consent (Attachment 1). The investigators read and explained the questionnaire to the subjects. The investigators measured the subjects’ MoCA score, height, weight, and finger tapping frequency.

### Montreal Cognitive Assessment (MoCA)

Mild cognitive impairment of participants was determined by MoCA, which is a 10 min, 30-point cognitive screening test designed to assist health professionals in the detection of MCI in patients scoring between 24 and 30 points on the MMSE. The MoCA included eight cognitive domains: attention and concentration, executive function, memory, language, visual skills, abstract thinking, structure calculation and directional force. The total score of the MoCA was 30 points. The MoCA was given to all the participants at baseline. The suggested cut-off point on the MoCA was 26. Participants with a score of 26 points or more were considered normal, meaning that the individual was without MCI. The score of the MoCA was slightly adjusted according to the level of education (if the subjects had an education duration of less than 12 years, a point was added to final the MoCA score). The MoCA is highly sensitive, involves a short testing time, covers important fields of cognition, and is suitable for clinical use. We calculated each subject’s MoCA score. Group one (controls) included 20 subjects with MCI and 20 healthy subjects without MCI. Group two (Tai Chi) included 30 subjects with MCI and 30 healthy subjects without MCI.

### Anthropometric Measurements

A variety of anthropometric measurements, including weight and height, were measured when the subjects were in light clothing without shoes. Weight was measured using electronic scales (Tanita Co., Japan) to the nearest 0.1 kg and height was measured to the nearest 0.1 cm.

### Finger Tapping Test

**FIGURE 1:**
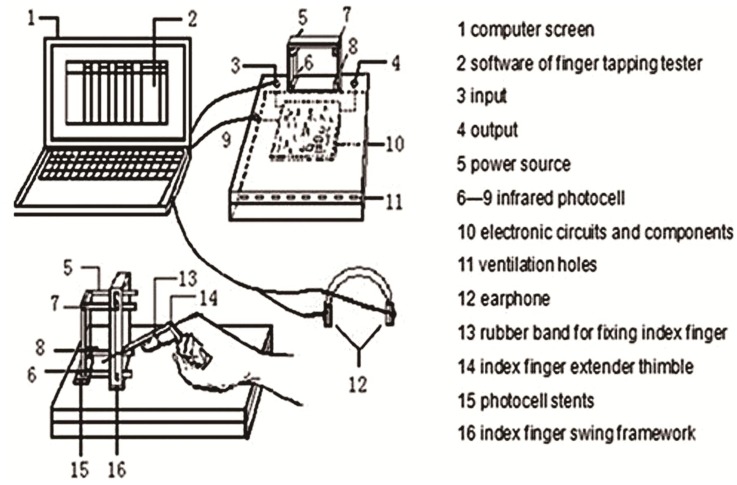
Sketch of the tester of finger tapping.

The examining instrument for finger tapping applied in the present study was granted a patent as well as the independent intellectual property right of China (**Figure 1**; Invention patent number: 200410017340.1). Finger tapping movements were detected with an infrared photoelectric sensor and related data were input into a computer through a serial port with a millisecond precision setting.

The index finger showed the fastest, most subtle and flexible reaction among all body parts. The data within the set time of 8 s were recorded. The subjects’ age, gender, major in sports, and result comparisons were included in the software design to provide a reasonable proof for the test results (Zhang L. et al., 2014). In order to reduce the effect of fatigue on the speed of forefinger tapping, the test time was set to 8 s.

Subjects were asked to sit with a normal posture during the test (head straight, eyes staring in front of the frame), metacarpophalangeal joint arch, the palm heel and three lateral fingers contacting the desktop, index finger extension stretch into the frame. Since there are individual differences in finger length, the swing angles differed in order to achieve the same oscillation amplitude.

Subjects started the formal test after 1–2 exercises. When the tester and the subject were both ready, the subject began to swing his/her index finger tap rapidly and repetitively as soon as he/she heard the starting gun and at the same time the instrument started timing automatically. The data were input into a computer within the 8 s set time.

### Statistical Analysis

SPSS 13.0 software (SPSS, Chicago, IL, United States) was applied to analyze the data, which were expressed as mean ± SD in Tables. Statistical differences were calculated using the independent samples *t*-test and two-Way ANOVA. *P*-values< 0.05 were considered statistically significant.

## Results

**Table 1** shows that the MoCA score of MCI subjects was significantly lower than that of healthy subjects without MCI (*P* < 0.01), which was not influenced by age, weight or height. **Table 2** shows that the finger tapping frequency of MCI subjects’ non-dominant left hand was significantly lower than that of healthy subjects without MCI (*P* < 0.01), and the trend of finger tapping frequency for the right hand was the same as the left hand.

**Table 1:** Information of the subjects from group one.

Group	*N*	Male	Female	Age (year)	Weight (kg)	Height (cm)	MoCA
Normal	20	7	13	62.0 ± 4.6	63.7 ± 10.5	160.3 ± 5.9	27.7 ± 1.3
MCI	20	7	13	63.0 ± 6.4	67.5 ± 10.5	160.1 ± 15.3	22.9 ± 1.8^∗∗^

*^∗^P < 0.05, ^∗∗^P < 0.01, vs. Normal.*

**Table 2:** Finger tapping of the subjects with and without MCI in group one.

Group	*N*	MoCA	Finger tapping of Left hand	Finger tapping of Right hand
Normal	20	27.7 ± 1.3	55.6 ± 14.1	62.2 ± 10.0
MCI	20	22.9 ± 1.8^∗∗^	40.1 ± 14.6^∗∗^	49.2 ± 12.3^∗∗^

*^∗^P < 0.05, ^∗∗^P < 0.01, vs. Normal.*

**Table 3** shows that the MoCA score of MCI subjects in the Tai Chi class was also significantly lower than that of healthy subjects without MCI (*P* < 0.01), and was not influenced by age, weight or height. **Table 4** shows that the finger tapping of MCI subjects’ dominant right hand was lower than that of healthy subjects without MCI in the Tai Chi class (*P* < 0.05), but no significant difference regarding the finger tapping frequency of the non-dominant left hand was observed.

**Table 3:** Information of the subjects in group two.

Group	*N*	Male	Female	Age (year)	Weight (kg)	Height (cm)	MoCA
Non-MCI (Taichi)	30	17	13	66.7 ± 2.7	60.5 ± 9.4	161.2 ± 9.6	28.0 ± 1.3
MCI (Taichi)	30	9	21	67.2 ± 7.9	60.5 ± 10.4	160.2 ± 6.0	22.7 ± 2.2^∗∗^

*^∗^P < 0.05, ^∗∗^P < 0.01, vs. Non-MCI (Taichi).*

**Table 4:** Finger tapping of the subjects with and without MCI in group two.

Group	*N*	MoCA	Finger tapping of Left hand	Finger tapping of Right hand
Non-MCI (Taichi)	30	28.0 ± 1.3	49.6 ± 14.4	58.4 ± 11.4
MCI (Taichi)	30	22.7 ± 2.2^∗∗^	43.1 ± 16.5	50.5 ± 14.6^∗^

*^∗^P < 0.05, ^∗∗^P < 0.01, vs. Non-MCI (Taichi).*

**FIGURE 2:**
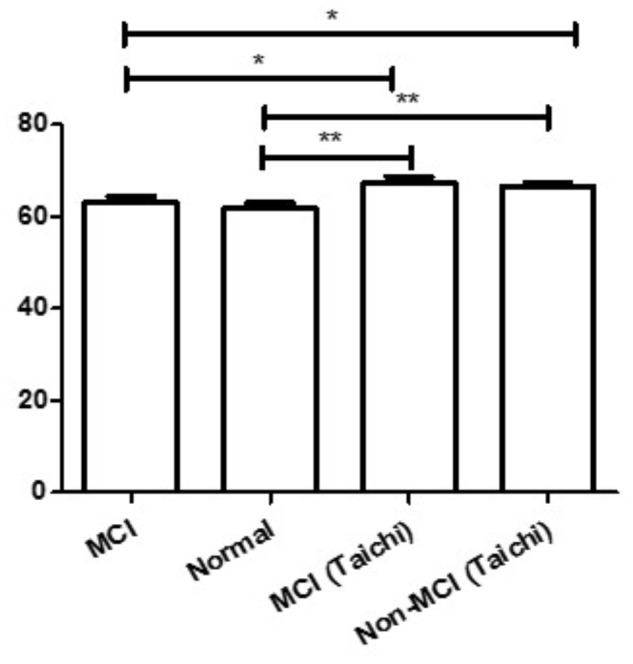
Comparison with subjects’ age of the four groups. ^∗^*P* < 0.05, ^∗∗^*P* < 0.01.

We calculated the four groups of age data using two-Way ANOVA. The results revealed that the age of MCI subjects was significantly lower than that of MCI subjects in the Tai Chi class (*P* < 0.05) and healthy subjects without MCI in the Tai Chi class (*P* < 0.05) (**Figure 2**). Also the age of healthy subjects without MCI was significantly lower than that of MCI subjects in the Tai Chi class (*P* < 0.01) and healthy subjects without MCI in the Tai Chi class (*P* < 0.01) from **Figure 2**.

## Discussion

Dementia is a syndrome of impaired cognitive functioning that interferes with the patient’s ability to live independently. It is a global public health issue (Liao et al., 2016). MCI is considered to be an intermediate clinical stage between normal cognitive aging and mild dementia. There is a considerable risk of MCI developing into dementia, but its progression over time varies tremendously among patients. AD is the most common diagnosis of dementia in elderly persons and is one of the devastating neurodegenerative disorders (An et al., 2016). AD is characterized by progressive loss of memory, thinking, learning, and cognitive ability, aggregation (including extracellular deposition) of the Aβ peptide, and intracellular aggregation of phosphorylated tau protein (Kim-Ha and Kim, 2016).

Since the pathology of AD develops slowly from a preclinical or early phase into a fully expressed clinical syndrome, the disease progression varies during diagnosis (Kitamura et al., 2017). MMSE and MoCA are commonly used for cognitive screening tests designed to assist health professionals in the detection of MCI in patients. But MoCA might play an important role in screening amnestic MCI and MCI patients with other cognitive impairments due to the better sensitivity of the MoCA and its wide coverage of multiple cognitive domains. We calculated the MoCA score of each subject.

Finger tapping is affected by many factors including hand dominance, age, gender, and neural control. Neuroimaging studies suggest that the primary motor area of the hand and the cerebellum plays a pivotal role in the control of finger tapping (Jancke et al., 2004), but different kinds of peripheral nerves in the upper limb contribute to this task, too. All the subjects in the present study were right-handed, and this was to ensure that the finger tapping test was not affected by differences in the dominant hand of subjects.

Firstly, we thought that the four groups of data could be analyzed using two-factor ANOVA, and the statistical differences were calculated using the independent sample *t*-test. But we calculated the age data of the four groups using two-Way ANOVA and found that there was an age difference. From **Figure 2**, the results revealed that the age of MCI subjects was significantly lower than that of both the MCI subjects and healthy subjects without MCI in the Tai Chi class (*P* < 0.05), and the age of the healthy subjects without MCI was also significantly lower than that of MCI subjects and healthy subjects without MCI in the Tai Chi class (*P* < 0.01). Age is an important factor that affects finger tapping. Age-related slowing of tapping has been found in many studies. Finger tapping frequency decreases with increasing age (Hubel et al., 2013). Age-related slowing might reflect an increased number of slow or incomplete taps (Cousins et al., 1998). Therefore, we compared the finger tapping frequency according to the groups mentioned in the material and methods. From **Tables 1**, 3, we found no significant difference in age, weight or height between subjects with MCI and subjects without MCI in both the groups, but the MoCA score of MCI subjects was significantly lower than that of healthy subjects without MCI.

From **Tables 2**, **4**, we found that the finger tapping frequency of MCI subjects’ non-dominant left hands was significantly lower compared to that of healthy subjects without MCI (*P* < 0.01), and a similar trend was observed for subjects’ dominant right hand. According to previous studies, MCI reduces finger tapping performance. Meanwhile, in group two, the finger tapping of MCI subjects’ dominant right hands was lower compared to that of healthy subjects in the Tai Chi class without MCI (*P* < 0.05); but no significant difference regarding the finger tapping frequency of the non-dominant left hand was observed between subjects with MCI and subjects without MCI.

These results suggest that finger tapping frequency of MCI subjects is significantly lower compared to normal subjects without MCI, and long-term Tai Chi exercise may increase the finger tapping frequency of MCI subjects toward normal levels. We found that Tai Chi reduced the significant difference in finger tapping frequency between healthy subjects and MCI patients. We also found that there was no difference in finger tapping frequency of the non-dominant left hand between healthy subjects and MCI patients.

The effect of MCI on the functional activities of patients in daily life is not easy to discern. A progression in the symptoms of MCI may occur after diagnosis in some patients, whereas a substantial proportion of patients do not develop dementia even after a prolonged period of up to 10 years (Visser et al., 2006). However, one way of discerning the effect of MCI on movement is through finger tapping frequency.

In healthy individuals, tapping rate is approximately 10% faster in the dominant hand than non-dominant hand (Ashendorf et al., 2009), and with extended tapping there is less fatigue in the dominant than in the non-dominant hand (Hubel et al., 2013). Differences between hands have also been observed in tap rate regularity (greater in the dominant hand), tapping downtime (reduced in the dominant hand), and applied force (smaller variance in applied up and down force in the dominant hand) (Hubel et al., 2013). For most individuals the frequency of useage is less in the left hand, the non-dominant hand, than the dominant hand in daily life. Since the non-dominant hand is used less in normal activities it is relatively untrained, and thus the performance of the non-dominant hand could better reflect the effects of Tai Chi exercise. Previous studies have found that after 2 weeks of rehabilitation treatment, motor function of the unaffected hand of stroke patients was obviously improved (Zhang L. et al., 2014). Thus, we would expect that motor function of the unaffected hand would be improved by long-term exercise such as Tai Chi, rehabilitation treatment, and so on.

Tai Chi is a kind of traditional Chinese boxing, and a form of low- to moderate-intensity mind-body exercise. It is a form of body-mind fitness that has a long history in the East (Zhang J. et al., 2014). Tai Chi can impart a protective effect against cognitive decline in aging and AD. For example, there are long-term health-promoting and protective effects of Tai Chi exercise, including the potential to induce neuronal and vascular plasticity in aging (Yeh et al., 2008; Nguyen and Kruse, 2012; Du et al., 2015).

In this study, we found that the finger tapping frequency of MCI subjeng frequency of MCI subjects is significantly lower than that of normal subjects without MCI, and long-term Tai Chi exercise increased the finger tapping frequency of the non-dominant hand of MCI subjects. We propose that a finger tapping test could detect subtle deficits in movement speed of patients with MCI, and this would allow an early diagnosis of the disorder. In this way, patients with an early MCI diagnosis could receive further examination and treatment. Tai Chi exercise is one treatment possibility that may reduce finger frequency movement deficits in MCI.

## Author Contributions

JZ and LZ designed this study. YZ, CS, LL, JD and DZ performed the experiments. LZ analyzed experimental data and also was responsible for manuscript writing. MW revised the manuscript. All authors approved the final version of this manuscript.

## Conflict of Interest Statement

The authors declare that the research was conducted in the absence of any commercial or financial relationships that could be construed as a potential conflict of interest.
